# Screening and identification of LMNB1 and DLGAP5, two key biomarkers in gliomas

**DOI:** 10.1042/BSR20210231

**Published:** 2021-05-21

**Authors:** Ding Zhou, Mengmeng Wang, Yu Zhang, Kai Wang, Min Zhao, Yan Wang, Xu Wang, Rutong Yu, Xiuping Zhou

**Affiliations:** 1 Institute of Nervous System Diseases, Xuzhou Medical University, Xuzhou, Jiangsu 221002, China; 2 Department of Neurosurgery, The Affiliated Hospital of Xuzhou Medical University, Xuzhou, Jiangsu 221002, China; 3 The Graduate School, Xuzhou Medical University, Xuzhou, Jiangsu 221002, China

**Keywords:** bioinformatics, DLGAP5, glioma, LMNB1, prognosis, proliferation

## Abstract

Glioma is the most common primary cancer in the central nervous system. Despite advances in surgery, radiotherapy and chemotherapy over the past decades, the prognosis of glioblastoma patients remains poor. We aim to identify robust gene signatures to better understand the complex molecular mechanisms and to discover potential novel molecular biomarkers for glioma. By exploring GSE16011, GSE4290 and GSE50161 data in Gene Expression Omnibus (GEO) database, we screened out 380 differentially expressed genes between non-tumor and glioma tissues, and further selected 30 hub genes through the Molecular Complex Detection (MCODE) plug-in in Cytoscape. In addition, LMNB1 and DLGAP5 were selected for further analyses due to their high expression in gliomas and were verified by using our cohort. Our study confirmed that LMNB1 and DLGAP5 were up-regulated in gliomas, and patients with high expression of LMNB1 or DLGAP5 had poor survival rate. Furthermore, silence of LMNB1 and DLGAP5 inhibited the proliferation of glioma cells. Together, LMNB1 and DLGAP5 were two potentially novel molecular biomarkers for diagnosis and prognosis of glioma.

## Introduction

Glioma is the most common primary cancer in the central nervous system. The 5-year survival rate of glioma patients is poor due to the invasive growth of the tumor and difficulty in its complete resection [1]. Glioblastoma (GBM) is the most malignant type of gliomas. Among the primary diagnosed GBM patients, almost 50% die within 1 year, while 90% die within 3 years [2]. Therefore, understanding the possible molecular mechanism of gliomas is a very important prerequisite for the discovery of novel and effective therapeutic strategies [3].

Recently, the high‐throughput sequencing and *in silico* technologies supplied powerful measures for cracking key genetic variations in many medical fields [4,5]. By using these novel technologies, researchers have discovered some molecular structures, molecular mechanisms, and novel biomarkers in a large number of cancers [6]. For example, the genes, such as RANBP17, ZNF734, NLRP2, GPR1, CCDC81, SH3RF1 and TM7SF4, were reported to be good prognostic biomarkers for GBM [7]. Geng et al. has discovered that several core biomarkers, such as TP53, TOP2A, CDK1, CCNB1, CDC20, CCNA2, NDC80, AURKA, BIRC5, CCNB2, KIF11 and MAD2L1, were associated with the outcome of glioma [8]. Therefore, bioinformatics is a good method to explore the potential diagnosis and prognosis biomarkers for gliomas for it can reduce the cost of study and provide a preliminary research idea.

The Gene Expression Omnibus (GEO), a comprehensive public database supported by National Center for Biotechnology Information, collects vast tumor gene expression profiles [9]. The GEO database is widely utilized to identify key genes related to cancer progression and prognosis, and to construct molecular regulatory network [10]. Despite numerous studies, no breakthrough has been made in the diagnosis and prognosis of gliomas. Therefore, discovering potential molecular biomarkers of glioma is critical to better understand its molecular signature and to improve its clinical outcomes. In the present study, based on the gene expression profiles in GEO database, we performed integrated bioinformatics analyses to probe the potential biomarkers for glioma. We used three GEO datasets and two authoritative glioma databases, including TCGA and CGGA, to find out key genes which play significant roles in glioma malignant progression. More importantly, we used the glioma specimens from the patients of the Affiliated Hospital of Xuzhou Medical University to verify the key genes we screened. Furthermore, we researched the roles of LMNB1 and DLGAP5 in glioma cell proliferation by silencing LMNB1 and DLGAP5.

## Materials and methods

### Microarray data of glioma

GEO (https://www.ncbi.nlm.nih.gov/geo/) is a public functional genomics database repository of high throughout gene expression data, chips and microarrays. All the gene expression in GSE16011 [11], GSE4290 [12] and GSE50161 [13] were downloaded from GEO. The platform of GSE16011 was GPL8542 (Affymetrix Gene Chip Human Genome U133 Plus 2.0 Array). The platform of GSE4290 and GSE50161 datasets was GPL570 (Affymetrix Human Genome U113 Plus 2.0 Array). GSE16011 dataset contained 159 glioma samples and 8 non-tumor samples. GSE4290 contained 77 glioma samples and 23 non-tumor samples, while GSE50161 contained 34 glioma samples and 13 non-tumor samples.

The data of TCGA [14] is from UCSC Cancer Genomics Browser-TCGA(Glioma) (http://genome-cancer.ucsc.edu) [15]. The gene expression profile was measured experimentally using the Illumina HiSeq 2000 RNA Sequencing platform by the University of North Carolina TCGA genome characterization center. Level 3 data were downloaded from TCGA data coordination center. This dataset shows the gene-level transcription estimates, as in log2(*x* + 1) transformed RSEM normalized count. The data of CGGA are from CGGA-mRNAseq-693 (the Chinese Glioma Genome Atlas, http://www.cgga.org.cn/index.jsp) [16]. The Platform for CGGA-mRNAseq-693 was Illumina HiSeq. Patients with higher or lower level than the median expression were named high or low group respectively.

### Identification and screening of differentially expressed genes

The differentially expressed genes (DEGs) between glioma and non-tumor samples were screened by GEO2R (https://www.ncbi.nlm.nih.gov/geo/geo2r/). As an interactive online tool, GEO2R can help researchers to compare multiple datasets in GEO series for identifying DEGs in different studies [17]. The adjusted *P*-values (adj. p, calculated by Benjamini and Hochberg method) were used to provide a balance between discovery of statistically significant genes and limitations of false-positives. Adj. *P*-value <0.01 and log fold change FC (logFC) >2 or < -2 were considered statistically significant.

### KEGG and GO enrichment analyses of DEGs

g: profiler(https://biit.cs.ut.ee/gprofiler/gost) [18] and The Database for Annotation, Visualization and Integrated Discovery (DAVID; http://david.ncifcrf.gov) [19] are two common online biological information data sets that integrate biological data and analysis tools and supply a series of functional annotation information of proteins or genes. KEGG is a common database integrated biological systems and senior functions which is produced by high-throughput sequencing technologies. As a classical bioinformatics tool, GO is able to gloss genes and analyze biological process of these genes [20,21].

### Construction of PPI network and module analysis

In our study, PPI network [22] of DEGs was constructed by STRING (http://string-db.org) (version 11.0) online database [23]. In addition, the interaction between each combined hub >0.4 was considered statistically significant. Cytoscape (version 3.7.1) is a common bioinformatics software to visualize molecular interaction in networks. The MCODE (version 1.5.1) [24] plug-in is an APP to cluster an existing network based on topology to discover densely connected regions. In MCODE plug-in, the standard for selection was as follows: MCODE score >5, degree cut-off = 2, node score cut-off = 0.2, Max depth = 100 and *k*-score = 2.

### The selection and analysis of hub genes

The biological process analyses of hub genes, which were selected with degrees ≥15, was shown and visualized by Biological Networks Gene Oncology tool (BiNGO) (version 3.0.3) plug-in [25]. The relationship between gene expression patterns and cancer grades and survival curve was analyzed by online database, Oncomine (http://www.Oncomine.com) [26], which collects all existing research data to verify other studies.

### Western blot

Protein was extracted from non-tumor or glioma tissues or cultured cells by whole cell lysis buffer. Forty micrograms of protein from each sample were separated by electrophoresis and transferred to polyvinylidene fluoride membranes (Millipore, MA), which was incubated overnight at 4°C with primary antibodies (LMNB1, 1:1000, A1910; DLGAP5, 1:1000, A13575, Abclonal; β-actin, 1:4000, ab8226, Abcam). Peroxidase‐conjugated affinipure goat anti-mouse IgG or anti-rabbit IgG (Biodragon; 1:4000) was used as the secondary antibody. Protein bands were measured by chemiluminescence ECL reagents (Thermo, Shanghai, China) and quantified using ImageJ software (Wisconsin) [27,28]. The samples (13 cases) collected from the Affiliated Hospital of Xuzhou Medical University were used in Western blot experiments. The use of the samples has been approved by the research ethics committee of the Affiliated Hospital of Xuzhou Medical University.

### Small interfering RNA transfection

For the siRNA transfection, cells were seeded in six-well plates at 50–70% confluence. LMNB1 and DLGAP5 siRNAs (siLMNB1 and siDLGAP5, 100 nM) or negative control (scramble, 100 nM) were transfected using Lipofectamine 2000, according to the protocol provided by the manufacturer. The LMNB1 and DLGAP5 siRNAs were synthesized by OBIO (Shanghai, China) and the sequences (5′ to 3′) were as follows:

siLMNB1-1: 5′-CAGAAAGAGUCUAGAGCAUTT-3′
siLMNB1-2: 5′-GCGAAGAUGUGAAGGUUAUTT-3′
siLMNB1-3: 5′-GCUUCUUGAUGUAAAGUUATT-3′
siDLGAP5-1: 5′-GCAAUGAGAGAGAGAAUUATT-3′
siDLGAP5-2: 5′-GGAUAUAAGUACUGAAAUGTT-3′
siDLGAP5-3: 5′-GAAAGAGCAGAGAGAGAAATT-3′

### EdU assay

The Cell-Light™ EdU Cell Proliferation Detection Kit (Ruibo Biotech, Guangzhou, China) was used to measure cell proliferation, according to the manufacturer’s instructions. Primary GBM and U251 cells were seeded into 96-well plates. After 24 h, 50 μΜ EdU was added and incubated with the cells for 2 h. The cells were then fixed with 4% paraformaldehyde for 15 min, followed by treatment with 0.5% Triton X-100 for 20 min. Subsequently, the cells were incubated with 1×Apollo® reaction cocktail for 30 min and stained with DAPI (Sigma) for 15 min. After having been washed for three times with PBS, the cells were subjected to imaging under a fluorescent inverted microscope. The experiment was performed three times.

### CCK-8 assay

Cell viability was examined using a Cell Counting Kit-8 (Dojindo, Kumamoto, Japan). GBM or U251 cells were seeded into 96-well plates (3000 cells per well). After having been cultured for the indicated hours, 10 μl of CCK-8 solution was added to each well. The absorbance at the OD450 nm was detected with a microplate reader after incubation for 2 h. The experiment was repeated three times.

### Cell cycle assay

Briefly, GBM and U87 cells were seeded into 6-cm dishes. The cells were collected and fixed with 70% ice-cold ethanol and subsequently washed twice with PBS. Cells were stained with 50 μg/ml propidium iodide (PI) solution containing 25 μg/ml ribonuclease (RNase) for 30 min. Finally, the cycle distribution was assessed by flow cytometry and analyzed using Cell Quest Pro software (Becton–Dickinson).

### Statistical analysis

Data were presented as mean ± standard error of the mean (SEM). Group differences were analyzed with the Student’s *t* test or one‐way analysis of variance (ANOVA). The software used for survival analyses was GraphPad Prism 7. Kaplan–Meier survival analyses was used to estimate the survival distribution, followed by a log‐rank test evaluating the difference between stratified groups, using the median value as the cutoff. *P*<0.05 was considered statistically significant.

## Results

### Identification of DEGs

**Figure 1:**
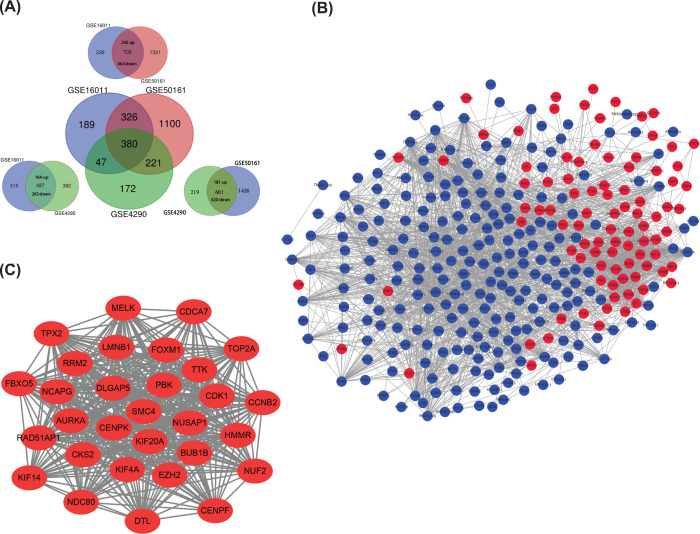
Screening of differential expression genes (DEGs) and extracting of hub genes (**A**) 380 DEGs abnormally expressed in glioma tissues were obtained from the intersection of GSE16011, GSE50161 and GSE4290. The DEGs from each two of the three datasets were shown in the surrounding. (**B**) PPI network formed by 380 genes. Red was the up-regulated genes and blue was the down-regulated ones. The minimum interaction score in STRING network was 0.4. (**C**) The hub gene network was analyzed by using MCODE for PPI network. Degree≥15, MCODE scores > 5, degree cut-off = 2, node score cut-off = 0.2, max depth = 100 and *k*-score = 2.

We analyzed non-tumor and glioma tissues in GSE16011, GSE4290 and GSE50161 by using the analysis function in GEO database. We set the logFC (|logFC|>2) and adj. *P* values (*P*<0.01) to further enhance the difference. As shown in Figure 1A, the Venn map exhibited 380 DEGs abnormally expressed in glioma tissues and co-existed in three GSE datasets, in which 109 genes were up-regulated and 271 genes were down-regulated. Thereafter, we performed the GO and KEGG analyses on David website and found that the main molecular functions of 380 genes were focused on gated channel activity (GO:0022836, FDR:5.60E-06), GABA receptor activity (GO:0016917, FDR: 9.15E-06) and substrate specific channel activity (GO:0022838, 3.83E-05). KEGG analyses indicated that DEGs were significantly enriched in morphine addition (hsa05032, FDR:3.97E-08), retrograde endocannabinoid signaling (hsa04723,FDR:2.05E-07) and nickel addition (hsa05033,FDR:3.35E-07) (Table 1)

**Table 1:** GO and KEGG pathway enrichment analysis of DEGs in glioma samples

Pathway ID	Description	Count in gene set	FDR
GO:0022836	Gated channel activity	27	5.60E-06
GO:0016917	GABA receptor activity	9	9.15E-06
GO:0022838	Substrate-specific channel activity	30	3.83E-05
GO:0015108	Chloride transmembrane transporter activity	14	4.73E-05
GO:0015267	Channel activity	31	5.02E-05
GO:0022803	Passive transmembrane transporter activity	31	5.27E-05
GO:0005216	Ion channel activity	29	6.50E-05
GO:0004890	GABA-A receptor activity	8	7.64E-05
GO:0005254	Chloride channel activity	13	1.07E-04
GO:0015276	Ligand-gated ion channel activity	16	3.37E-04
hsa05032	Morphine addiction	17	3.97E-08
hsa04723	Retrograde endocannabinoid signaling	17	2.05E-07
hsa05033	Nicotine addiction	12	3.35E-07
hsa04727	GABAergic synapse	15	1.79E-06
hsa04080	Neuroactive ligand-receptor interaction	20	0.004308636
hsa04020	Calcium signaling pathway	16	0.004437933
hsa04721	Synaptic vesicle cycle	10	0.0059572
hsa04728	Dopaminergic synapse	12	0.074662938
hsa04512	ECM-receptor interaction	10	0.084796959
hsa04724	Glutamatergic synapse	11	0.137170628

Abbreviations: FDR, false discovery rate; GO, Gene Ontology; KEGG, Kyoto Encyclopedia of Genes and Genomes.

### Construct PPI network and module analysis

We constructed the protein function network of the 380 gene representative proteins with STRING and Cytoscape, then generated PPI network (Figure 1B). Thereafter, we used MCODE plug-in to analyze the PPI network module and found the most densely linked 30 genes, including TTK, FBXO5, KIF20A, KIF4A, LMNB1, RRM2, DTL, RAD51AP1, CDCA7, MELK, CENPK, CCNB2, KIF14, AURKA, CDK1, BUB1B, DLGAP5, CKS2, NUF2, NDC80, SMC4, NCAPG, TPX2, EZH2, HMMR, NUSAP1, TOP2A, PBK, FOXM1 and CENPF. The interactive degree of these 30 hub genes was more than 15, and the results were shown in Figure 1C.

### Selection and analysis of the hub genes

**Figure 2:**
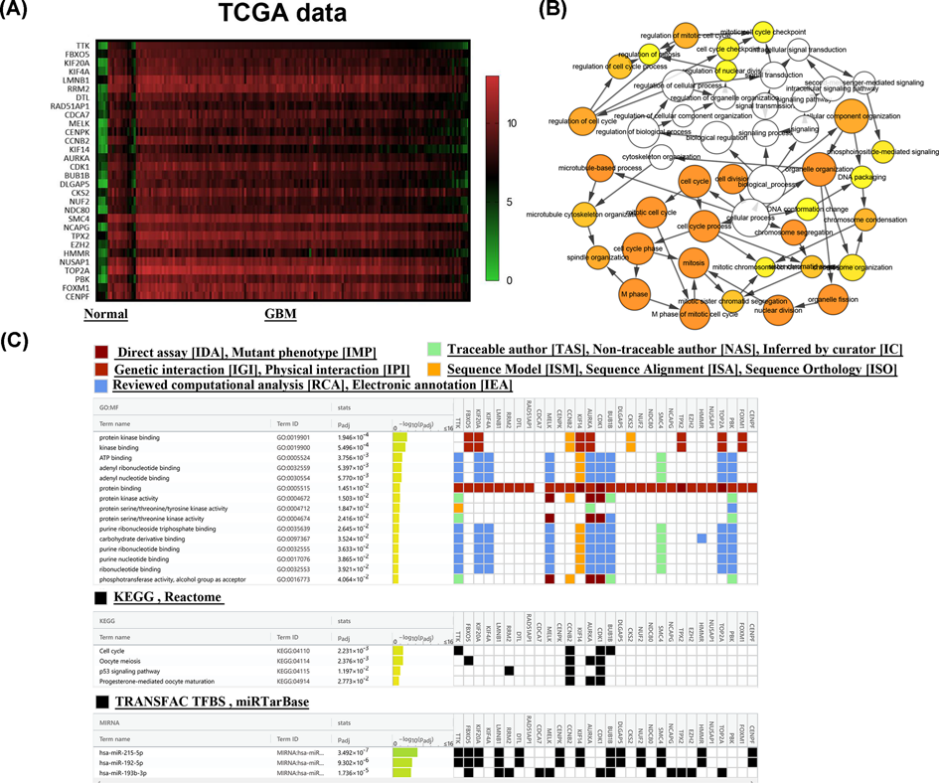
Analysis of the 30 hub genes (**A**) The 30 hub genes were up-regulated in TCGA GBM dataset. (**B**) The network of GO-biological process analyses of 30 hub genes. The circle size represented gene enrichment degree, and the depth of the color represented the size of the *P* value. (**C**) The GO-molecular function analyses, KEGG analyses and corresponding associated miRNAs of the hub genes. The color indicates different data sources and the data were arranged according to the size of *P* value. Result was from the website: g: profiler.

To further analyze these genes, we conducted a preliminary analysis of these pivotal genes in the TCGA database, which showed that these hub genes were indeed highly expressed in gliomas (Figure 2A). In addition, the biological process network of these genes was drawn by BiNGO plug-in (Figure 2B). Thereafter, we performed GO and KEGG analyses of these 30 hub genes and identified the relationship of these 30 hub genes with miRNAs on g: profiler. GO analyses showed that the molecular functions of these 30 hub genes were significantly enriched in protein kinase binding (GO:0019901), kinase binding (GO:0019900) and ATP binding (GO:0005524). The outcomes of KEGG analyses indicated that they were concentrated in cell cycle (GO:04110), oocyte meiosis (GO:04114), p53 signaling pathway (GO:04115) and progesterone-mediated oocyte maturation (GO:049114). Interestingly, these hub genes were known to be regulated by miRNAs, such as hsa-miR-215-5p [29], hsa-miR-192-5p [30,31] or hsa-miR-193b-3p [32,33], which were related to the development of cancer (Figure 2C).

### High expression of LMNB1 and DLGAP5 in gliomas

**Figure 3:**
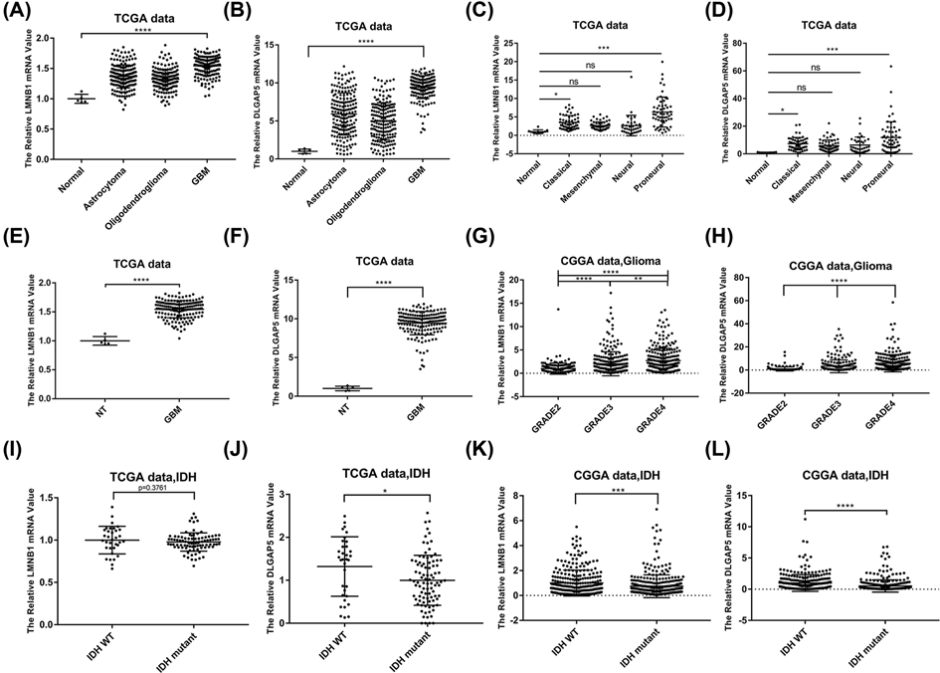
Expression of LMNB1 and DLGAP5 in TCGA and CGGA databases (**A **and** B**) Expression of LMNB1 and DLGAP5 in different glioma cell types in TCGA database. (**C **and** D**) Expression of LMNB1 and DLGAP5 in different glioma molecular subtype in TCGA database. (**E **and** F**) Expression of LMNB1 and DLGAP5 in non-tumor tissues and GBM in TCGA database. (**G **and** H**) Expression of LMNB1 and DLGAP5 in different grade of gliomas in CGGA database. (**I–L**) Expression of LMNB1 and DLGAP5 in gliomas with or without IDH mutation in TCGA (I and J) and CGGA database (K and L); *P<0.05, **P<0.01, ***P<0.001, ****P<0.0001, with one‐way ANOVA, NT: non-tumor.

Among the above selected 30 genes, we found that LMNB1 and DLGAP5 were highly expressed but rarely studied in gliomas, indicating great research potential. Therefore, we focused our work on these two genes. We analyzed the expression of LMNB1 and DLGAP5 in glioma patients with different histological subtype (Figure 3A,B), molecular subtype (Figure 3C,D), progression stage (Figure 3E–H) and IDH status (Figure 3I–L) in TCGA and CGGA databases. In TCGA database, we found that LMNB1 and DLGAP5 were highly expressed in oligodendrogliomas, astrocytoma and GBM. In addition, the GBM exhibited the highest expression level of LMNB1 and DLGAP5 (Figure 3A,B). Among four glioma subtypes (proneural, mesenchymal, classical and neural), the proneural subtype exhibited the highest expression level of LMNB1 and DLGAP5, and the neural subtype expressed the lowest levels (Figure 3C,D). Similarly, Grade IV gliomas showed the highest LMNB1 and DLGAP5 expression level in CGGA RNAseq datasets (Figure 3G,H). Furthermore, IDH1 wild-type patients had higher level of LMNB1 and DLGAP5 than that of IDH1 mutant patients (Figure 3I–L). Together, these data indicated that LMNB1 and DLGAP5 were up-regulated in glioma and might play specific roles in different molecular subtype.

### High expression of LMNB1 and DLGAP5 correlates with worse prognosis

**Figure 4:**
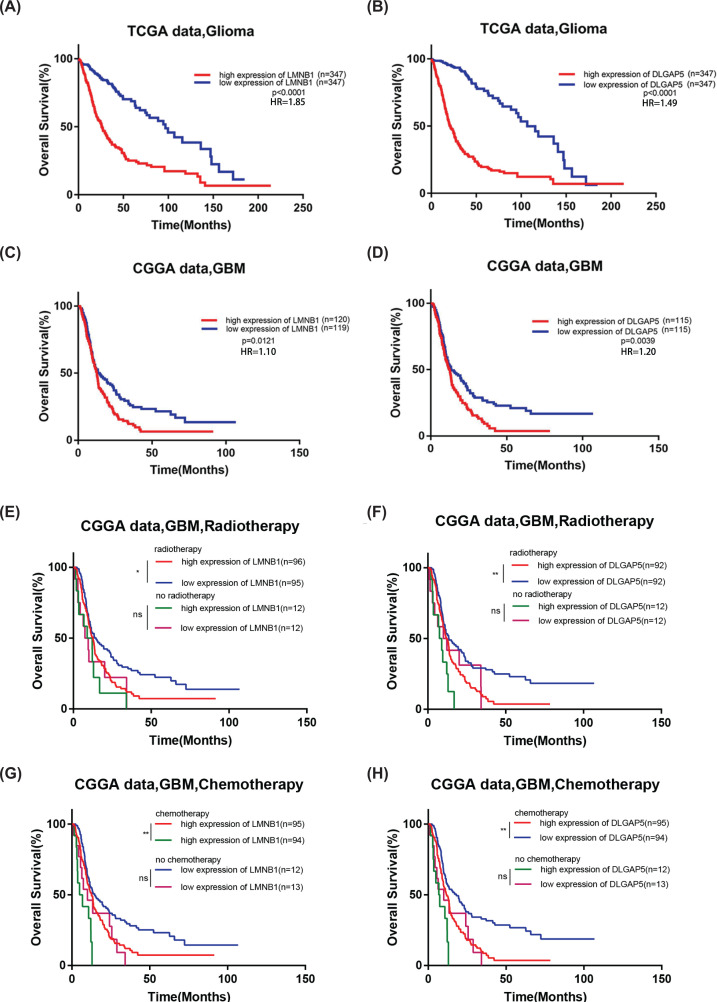
The correlation of LMNB1 and DLGAP5 expression with patient overall survival time in TCGA and CGGA databases (**A **and** B**) High expression of LMNB1 or DLGAP5 exhibited worse overall survival curve of glioma patients in TCGA database. (**C **and** D**) High expression of LMNB1 or DLGAP5 exhibited worse overall survival curve of glioma patients in CGGA database. (**E **and** F**) Overall survival curve of GBM patients divided by expression of LMNB1 or DLGAP5 with or without radiotherapy in CGGA database. (**G **and** H**) Overall survival curve of GBM patients divided by expression of LMNB1 or DLGAP5 with or without chemotherapy in CGGA database; *P<0.05, **P<0.01, ***P<0.001.

We further analyze the correlation of these two genes with the survival rate of patients with different subtype, including tumor type (Figure 4A–D), radiotherapy (Figure 4E,F) and chemotherapy status (Figure 4G,H). Patients with higher or lower level than the median expression were assigned to high or low group respectively. The analysis results showed that glioma patients with high expression of LMNB1 and DLGAP5 exhibited worse overall survival rate (Figure 4A–D). Similar results were found in chemotherapy and radiotherapy group (Figure 4E–H) in CGGA database, in which GBM patients with high expression of LMNB1 and DLGAP5 displayed worse survival rate. In conclusion, these results showed that LMNB1 and DLGAP5 may be used to predict the prognosis of glioma patients.

### High expression of LMNB1 and DLGAP5 in oncomine database and clinical samples

**Figure 5:**
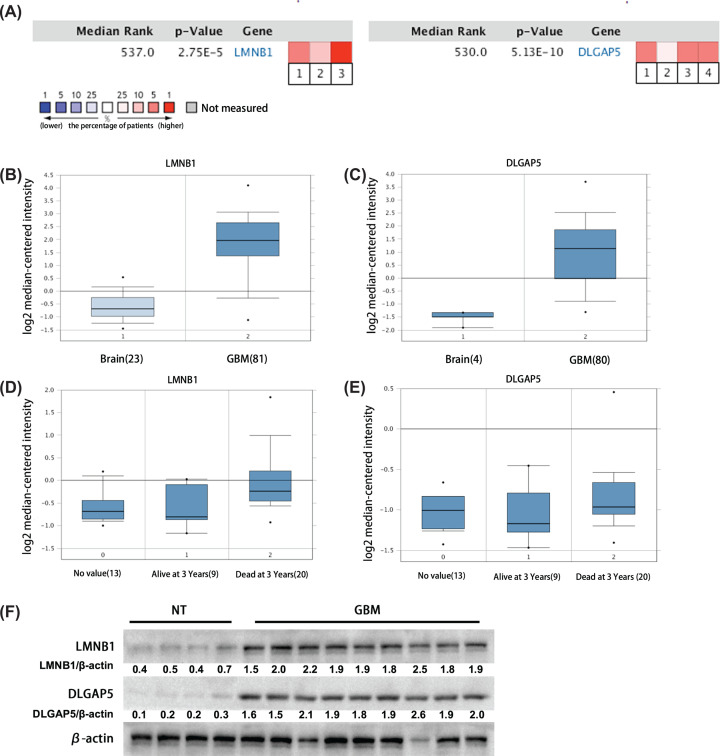
The expression of LMNB1 and DLGAP5 in Oncomine database and clinical specimens (**A**) LMNB1 (left) increased in glioma group reported in three previous studies, and DLGAP5 (right) increased in glioma group reported in four previous studies (non-tumor vs. glioma 1. Murat Brain, J. Clin. Oncol., 2008. 2. Shai Brain, Oncogene, 2003. 3. Sun Brain, Cancer Cell, 2006. 4. TCGA Brain, No Associated Paper, 2013.) Blue indicated the percentage of patients with lower nucleic acid level of the gene. Red indicated the percentage of patients with higher expression of the gene. (**B **and** C**) The specific expression of LMNB1 and DLGAP5 in the database. (Left) Sun Brain, Cancer Cell, 2006. (Right) Murat Brain, J. Clin. Oncol., 2008. (**D **and** E**) The expression of LMNB1 and DLGAP5 in patients dead or survival at 3 years after primary glioma diagnosis in previous studies. The results showed that the level of LMNB1 and DLGAP5 in patients dead at 3 years after primary glioma diagnosis was higher than that of alive patients (Shai Brain, Oncogene, 2003); *P<0.05, **P<0.01. (**F**) The protein level of LMNB1 and DLGAP5 in non-tumor and GBM clinical specimens examined by Western blot. Numbers meant the quantified protein level of LMNB1 and DLGAP5.

Oncomine is an online database, which collects all existing research data and is extensively used to verify other studies. We thus analyzed these two genes again in Oncomine database and found that high expression of LMNB1 in glioma tissues was reported in three studies, and high expression of DLGAP5 was reported in four studies (Figure 5A). Compared with non-tumor tissues, LMNB1 and DLGAP5 were highly expressed in glioma tissues, which was reflected in the research of Sun [12] and Murat [34] (Figure 5B,C). Moreover, according to the study of Shai [35], the expression level of LMNB1 and DLGAP5 was lower in patients who survived longer than 3 years but higher in patients who died within 3 years after diagnosis (Figure 5D,E).

In addition, we also detected the protein level of LMNB1 and DLGAP5 in GBM clinical samples collected from the Affiliated Hospital of Xuzhou Medical University (Figure 5F). The results showed that both LMNB1 and DLGAP5 were highly expressed in GBM tissues indeed, in line with the online database analysis.

### Silence of LMNB1 or DLGAP5 inhibits the proliferation of glioma cells

**Figure 6:**
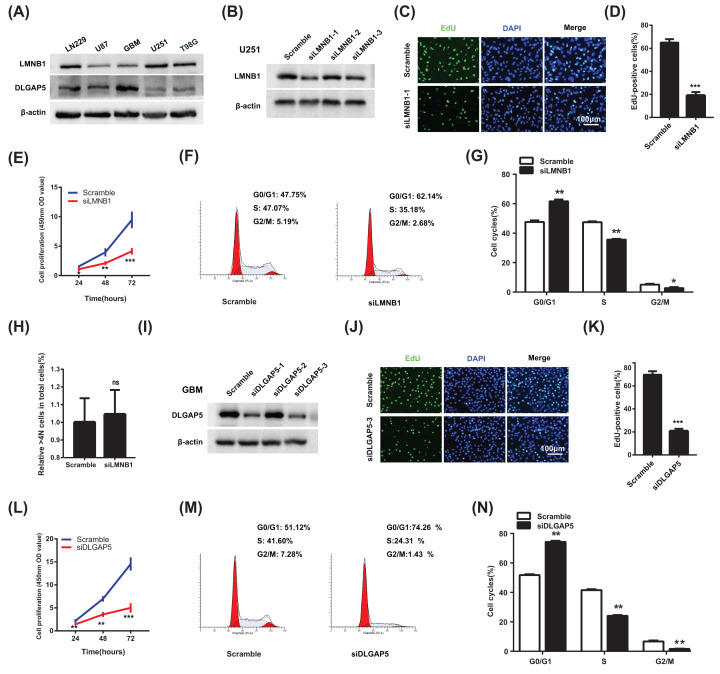
Silence of LMNB1 and DLGAP5 inhibit the proliferation of glioma cells (**A**) Basal protein levels of LMNB1 and DLGAP5 in glioma cell lines were examined by Western blot. (**B**) Down-regulation effect of LMNB1 siRNAs was checked in glioma cells. (**C **and** D**) The effect of siLMNB1-1 on glioma proliferation was examined by EdU assay; scale bar: 100 μm. (**E**) The effect of siLMNB1-1 on glioma cells viability was examined by CCK-8 assay. (**F **and** G**) The representative images (F) and quantification results (G) of flow cytometry after siLMNB1-1 transfection. (**H**) Percentage of cells with DNA content >4N in total cells with siLMNB1-1 transfection. (**I**) Down-regulation effect of DLGAP5 siRNAs was checked in glioma cells. (**J **and** K**) The effect of siDLGAP5-3 on glioma proliferation was examined by EdU assay; scale bar: 100 μm. (**L**) The effect of siDLGAP5-3 on glioma cells viability was examined by CCK-8 assay. (**M **and** N**) The representative images (M) and quantification results (N) of flow cytometry after siDLGAP5-3 transfection. The results were representative of experiments repeated at least 3 times and presented as mean ± SEM; *P<0.05, **P<0.01, ***P<0.001; ns, no significance.

Since both of LMNB1 and DLGAP5 were up-regulated in glioma tissues, we therefore down-regulated LMNB1 and DLGAP5 with siRNAs to detect whether silence of LMNB1 or DLGAP5 inhibits the proliferation of glioma cells. First, we detected the basal protein level of LMNB1 and DLGAP5 in several glioma cell lines including LN229, U87, primary cultured GBM cell (GBM), U251, T98G. The result showed that U251 cell exhibited relatively high LMNB1 and GBM cell exhibited relatively high DLGAP5 (Figure 6A). We therefore chose U251 cell and GBM cell to down-regulate LMNB1 and DLGAP5 respectively in the subsequent experiments. As is shown in Figure 6B, the down-regulation effect of siLMNB1-1 was the highest one among three siRNAs, which was chose to perform the following experiments. Examined by EdU and CCK-8 assay, we discovered that the percent of EdU positive cells and cell proliferation of glioma cells decreased after down-regulation of LMNB1 (Figure 6C–E). In addition, silence of LMNB1 arrested the cell cycle of glioma cells at G1 phase (Figure 6F,G), but did not affect the percentage of >4N cells in total cells (Figure 6H). Our data showed that down-regulation of LMNB1 inhibited the proliferation of glioma cells by arresting the cell cycle at G0/G1 phase.

Similarly, we chose siDLGAP5-3, which exhibited the highest silencing efficiency on DLGAP5 (Figure 6I), to examine the roles of DLGAP5 on glioma proliferation. Examined by EdU assay and CCK-8 assay, we found that silence of DLGAP5 inhibited the proliferation of glioma cells (Figure 6J–L). In addition, examined by flow cytometry, we found that, after DLGAP5 down-regulation, the cells in G0/G1 phase increased significantly, while the cells in S and G2/M phase decreased (Figure 6M,N). This result showed that down-regulation of DLGAP5 arrested the cell cycle of GBM cells at G0/G1 phase.

## Discussion

In recent years, with the development of high-throughput sequencing and microarray technology, bioinformatics measures have been widely used to find potential biomarkers for the diagnosis, prognosis, and even treatment of various tumors [38,39]. However, up to now, the analysis of the high-throughput datasets of glioma remains incomplete. In our research, GSE16011, GSE4290 and GSE50161 microarray datasets were selected to analyze DEGs between non-tumor and glioma tissues.

The integrated analyses revealed 380 intersecting genes abnormally expressed in glioma tissues. According to the significant network module from PPI network, 30 hub genes including TTK, FBXO5, KIF20A, KIF4A, LMNB1, RRM2, DTL, RAD51AP1, CDCA7, MELK, CENPK, CCNB2, KIF14, AURKA, CDK1, BUB1B, DLGAP5, CKS2, NUF2, NDC80, SMC4, NCAPG, TPX2, EZH2, HMMR, NUSAP1, TOP2A, PBK, FOXM1 and CENPF were filtered out, which were all significantly up-regulated in glioma samples by analyzing the TCGA and CGGA database. The KEGG pathway enrichment indicated substantial involvement of hub genes in cell cycle, oocyte meiosis, p53 signaling pathway and progesterone-mediated oocyte maturation (Figure 2). To our knowledge, cell cycle is closely associated with the occurrence or development of tumor [40] and p53 pathway is a very important pathway for various tumor progression [41], indicating that the hub genes we selected may associate with tumor progression. Indeed, by searching on PubMed, we found that most of them had a great impact on tumor. For example, Melk can enhance the bortezomib resistance of natural killer/T-cell lymphama through EZH2 [42], while the infection of CDK1 expression and activity reduced ovarian cancer growth [43]. The cancer promoting effect of HMMR in glioma has also been reported [44]. Microtubule nucleating factor (TPX2) plays an important role in mitotic spindle assembly [45]. Kinesin family member 4a (KIF4A), a kind of microtubule-based motor protein, is involved in maintaining chromosome integrity during cell mitosis [46].

After searching for TCGA and CGGA datasets, we found that the expression of LMNB1 and DLGAP5 were up-regulated in glioma. Consistently, we also confirmed this result by using our clinical samples (Figure 5F). Further analyses showed that the level of LMNB1 and DLGAP5 were higher in proneural subtype and IDH1 wild-type patients, indicating that LMNB1 and DLGAP5 may play specific roles in different molecular subtype of GBM. In addition, high LMNB1 and DLGAP5 expression patients showed shorter overall survival rate, indicating that LMNB1 and DLGAP5 have an important impact on the prognosis of patients. Consistent with the online analyses, we found that down-regulation of LMNB1 and DLGAP5 with siRNA inhibited the proliferation of glioma cells (Figure 6). Interestingly, in CGGA database, patients with high level LMNB1 and DLGAP5 showed poor prognosis after accepting radiotherapy or chemotherapy. The effect of LMNB1 and DLGAP5 on GBM survival was similar to that of OSMR [47]. Therefore, LMNB1 and DLGAP5 may mediate the radiotherapy or chemotherapy resistance in GBM patients.

According to the literature of Izdebska et al., overexpression of LMNB1 can induce mitotic mutations after 5-FU treatment, which is indexed by the percentage of >4N polyploid cells, in LoVo colon cancer cell [36]. However, in our system, we did not observe the increase of the percentage of >4N polyploid cells in glioma cells by flow cytometry (Figure 6H), indicating that silencing LMNB1 could not induce mitotic mutation. Since Izdebska et al. reported that overexpression of LMNB1 could enhance the percentage of >4N polyploid cells after 5-FU treatment, while it could not without 5-FU treatment [36], it suggests that the effect of LMNB1 on mitotic mutation was closely related to 5-FU treatment. In our system, we just down-regulated LMNB1 with siRNA without any other treatment. We therefore deduce the difference of result between our study and that of Izdebska et al. was caused by the different experimental parameter. In addition, Rolyan et al. has reported that overexpression of LMNB1 causes autosomal dominant leukodystrophy (ADLD). The mice with LMNB1 overexpression showed severe vacuolar degeneration of the spinal cord white matter, together with marked astrogliosis, microglial infiltration and secondary axonal damage, which is caused by abnormal lipid metabolism [48]. They further discovered that overexpression of LMNB1 resulted in the decrease of genes including ELOVL7, DHCR7, SC4MOL and so on, which is involved in lipid synthesis pathways. It will be interesting to determine whether LMNB1 promotes glioma progression via regulating lipid synthesis in the future.

DLGAP5, also known as DLG7 or HURP, is a mitotic spindle protein and the function of DLGAP5 is to promote the formation of tubulin polymers [49]. As a cell cycle regulated gene, the expression level of DLGAP5 mRNA has been proved to change periodically during the cell cycle and reaches a peak at the M phase in hepatocellular carcinoma cells by Tsou et al. [37]. They, therefore, guess that DLGAP5 regulates the M phase progression of hepatocellular carcinoma cells. However, Tsou et al. did not detect the cell cycle of hepatocellular carcinoma cell directly after down-regulation of DLGAP5. In our study, examined by flow cytometry, we found that, after DLGAP5 down-regulation, the cells in G0/G1 phase increased, while those in S and G2/M phase significantly decreased (Figure 6M,N). Our work indicated that DLGAP5 may promote glioma progression by speeding the G0/G1 phase progression.

Taken together, our analyses showed that LMNB1 and DLGAP5 were highly expressed in glioma and negatively associated with patient survival, indicating that LMNB1 and DLGAP5 were important for the development and prognosis of glioma. Therefore, these two genes deserve further study to explore the molecular mechanism in glioma progression and to identify its therapeutic potential.

## Clinical significance

1. LMNB1 and DLGAP5 were up-regulated in gliomas.
2. Silence of LMNB1 and DLGAP5 inhibited the proliferation of glioma cells.
3. LMNB1 and DLGAP5 were two potentially novel molecular biomarkers for diagnosis and prognosis of glioma.
4. High expression of LMNB1 and DLGAP5 may be involved in the therapeutic resistance of radiotherapy and chemotherapy.

## Data Availability

The data underlying this study are freely available from GEO database (https://www.ncbi.nlm.nih.gov/geo/), UCSC-TCGA (http://genome-cancer.ucsc.edu) and CGGA (http://www.cgga.org.cn/index.jsp). The authors did not have special access privileges

## Abbreviations

ADLD: autosomal dominant leukodystrophy
DEG: differentially expressed gene
FDR: false discovery rate
GEO: Gene Expression Omnibus
GO: Gene Ontology
KEGG: Kyoto Encyclopedia of Genes and Genomes
KIF4A: kinesin family member 4a
